# Microencephaly in fetal piglets following *in utero* inoculation of Zika virus

**DOI:** 10.1038/s41426-018-0044-y

**Published:** 2018-03-29

**Authors:** P. J. Wichgers Schreur, L. van Keulen, D. Anjema, J. Kant, J. Kortekaas

**Affiliations:** Wageningen Bioveterinary Research, Houtribweg 39, 8221 RA Lelystad, The Netherlands

## Abstract

Zika virus (ZIKV) is a mosquito-borne flavivirus that became associated with microcephaly in newborns and Guillain–Barré syndrome in adults after its emergence in the Pacific and the Americas in 2015. Newly developed rodent and nonhuman primate models have already revealed important insights into ZIKV-induced neuropathology. Nonhuman primates are phylogenetically closely related to humans and are therefore preferred human surrogates in ZIKV research. However, the use of nonhuman primates, particularly during gestation, raises ethical issues. Considering that pigs also share many anatomical and physiological features with humans, this species may be an attractive alternative human surrogate for ZIKV research. Here, we inoculated 20 porcine fetuses *in utero* and assessed the effect of ZIKV on brain development 4 weeks later. All inoculated fetuses presented mild to severe neuropathology, characterized by a depletion of neurons in the cerebral cortex. In most cases, neuronal depletion was confined to specific cerebral lobes without affecting brain size, whereas in severe cases a more generalized depletion resulted in microencephaly. Although the virus was widespread in the sows’ placenta at the time of necropsy only low levels of viral RNA were detected in fetal brain samples, thereby preventing the identification of primary target cells. Our findings suggest that pigs can be used to study ZIKV-induced neurodevelopmental defects as currently observed in human neonates, varying from stunted brain growth to localized cortical neuronal depletion in the absence of major macroscopic abnormalities.

## Introduction

Zika virus (ZIKV) is a mosquito-borne flavivirus that was for the first time isolated in 1947 from the blood of a rhesus monkey from the Zika forest in Uganda^1^. The ability of the virus to spread efficiently among humans became clear in 2007 when the virus caused an epidemic on the island of Yap^2^. In succeeding years, ZIKV caused additional epidemics on Pacific islands and spread to Brazil in 2015, where it initiated an unprecedented outbreak. During this outbreak, the virus was for the first time associated with microcephaly in newborns and Guillain–Barré syndrome in adults. The outbreak triggered the development of several rodent and nonhuman primate models, which already have proven their value by contributing significantly to the understanding of ZIKV-mediated neuropathology^3–5^.

Important insights into ZIKV-mediated neuropathology, including the identification of neuronal progenitor cells as primary targets of the virus, came from studies with mice^6–14^. Although mouse models will undoubtedly continue to prove their value in ZIKV research, the major differences in the physiology, anatomy, and development of the rodent and human brain makes extrapolation of observations made in mouse experiments to humans not always appropriate. The lower complexity of the mouse brain is macroscopically exemplified by its structure, which is lissencephalic (smooth), whereas the brains of most larger animals are gyrencephalic, containing deep folds with ridges (gyri) and depressions (sulci). The application of mouse models to study the effect of ZIKV on brain development is further limited by the short gestation period of mice compared with humans (20 vs. 280 days), prohibiting studies on less acute effects of ZIKV infection on brain development.

Nonhuman primates, such as rhesus macaques, are considered the best human surrogates for studying human infectious diseases and have indeed proven extremely valuable in ZIKV research^15–23^. However, the use of nonhuman primates, especially large numbers of pregnant animals, is under severe societal scrutiny. Moreover, only few institutions have the capacity to perform experiments with these animals. Another species that shares genetic, physiologic, immunologic, and anatomical features with humans and that is more commonly available is the domestic pig (*Sus scrofa domesticus*). Consequently, pigs are extensively used in studies on human infectious diseases and in studies on human brain disorders^24, 25^. Another advantage of pigs is that they carry many fetuses, thereby facilitating studies on prenatal development with multiple genetically related fetuses at the same time.

The susceptibility of newborn piglets to ZIKV infection was recently reported^26^. In this previous study, ZIKV was inoculated via intradermal, intraperitoneal (IP), or intracerebral route into newborn piglets. This preliminary, yet important study demonstrated that ZIKV can infect and replicate in piglets and that direct administration of the virus into the brain results in neurological disease that manifested with leg weakness, ataxia, and tremor. However, this study also suggested that the virus is not capable of autonomously gaining access to the central nervous system of newborn piglets. In the present work, we have investigated if ZIKV can cross the porcine placenta and cause congenital defects in fetal piglets.

Our results suggest that ZIKV does not cross the porcine placenta. However, when the placenta was bypassed, ZIKV infection resulted in serious neuropathological manifestations resembling those observed in humans. The resulting model is highly robust and can be used to study the effects of ZIKV infection on brain development, as well as associated cognitive and behavioral impairments.

## Materials and methods

### Ethics statement

The animal experiment was conducted in accordance with the Dutch Law on Animal Experiments (Wet op de Dierproeven, ID number BWBR0003081) and the European regulations (EU directive 2010/63/EU) on the protection of animals used for scientific purposes. The procedures were approved by the Animal Ethics Committee of Wageningen Bioveterinary Research (WBVR) and the Dutch Central Authority for Scientific Procedures on Animals (CCD). The CCD permit number is AVD401002016615 and the WBVR experiment licence number is 2016.D-0123.001. We received consent from the pig breeder to perform this experiment.

### Cells and viruses

ZIKV strain PRVABC59, originally isolated from human serum in Puerto Rico in 2015, was obtained from ATCC (VR-1843). This American strain of Asian lineage was isolated at the beginning of the current epidemic and was shown to cause disease in various animal models. A working stock was prepared after inoculation of the virus at low multiplicity of infection (0.01) on Vero-E6 cells (ATCC CRL-1586), which were grown in minimal essential medium supplemented with Earle’s salts (Invitrogen), 5% fetal bovine serum (Bodinco), 1% l-glutamine and 1 × antibiotic-antimycotic (Invitrogen). Virus titers were determined by incubating Vero-E6 cells, in 96-wells plates, with serial dilutions of the virus for 5–7 days using cytopathic effect as a readout.

### Animal experiment

At a registered pig breeder in the Netherlands, six Landrace sows, aged 1.5–4 years, who had delivered at least two healthy litters previously, were artificially inseminated after estrus synchronization, using sperm of a Landrace boar. After pregnancy was confirmed by ultrasound at 45 days post insemination, the sows were moved to a Biosafety Level-3 facility. Sows were randomly assigned to three groups of two animals and allowed to acclimatize for 5 days before the start of the experimental period. At day 0, sows of group I (numbers 1543 and 1544) were subjected to laparotomy. Briefly, sows were anesthetized using azaperon (Stressnil^R^, Elanco, 40 mg/ml, dose IM 1 ml/20 kg) as a premedication drug followed by intravenous (IV) (ear catheter) induction with propofol (Propofol^R^, Abbott, 10 mg/ml, dose IV 0.10–0.15 mg/kg) and analgesia using buprenorphine (Buprecare^R^, AST farma, 0.3 mg/ml, dose: IV 4–6 µg/kg). After placement of the sow onto a steel frame covered with a 10 cm thick isolating cushion, the sow was intubated and connected to a Datex-Ohmeda S/5 ADU care station (GE Healthcare, O_2_ 2–3 liter/min, air with Sevoflurane 2–4% [2–3 liter/min], AbbVie). After performing local anesthesia at the incision site at the left flank, using lidocaine hydrochloride (Lidocaine^R^, Dechra, intramuscular (IM), 80–100 ml) a 20–25 cm incision was made to gain access to the left uterine horn in the abdomen. Parts of the horn, starting from the ovary, were brought outside the animal and after localization of a fetus, a purse string suture was placed on the outside of the uterus wall and tightened after IP inoculation of the fetus with 10^5.6^ TCID_50_ (in 0.1 ml) of ZIKV strain PRVABC59. In total, 20 fetuses were inoculated: 9 in the left uterine horn of sow 1543 and 11 in the left uterine horn of sow 1544. The fetuses in the right uterine horns remained untreated to be able to assess potential virus transmission between uterine horns. After putting 1–1.5 liter physiological salt into the abdominal cavity to facilitate organ repositioning, the operation wound was closed. Sows were subsequently provided with 3000 mg paracetamol (rectal) per animal per day for 3 days. Additionally, sows were treated for 2 days with procaïnebenzylpenicilline (Depocilline^R^, MSD Animal Health, 15 ml, IM) to prevent bacterial infections. Of the *in utero* (IU)-inoculated animals, sow 1543 quickly recovered from surgery, whereas sow 1544 was less mobile and had reduced appetite for a few days before full recovery.

**Fig. 1:**
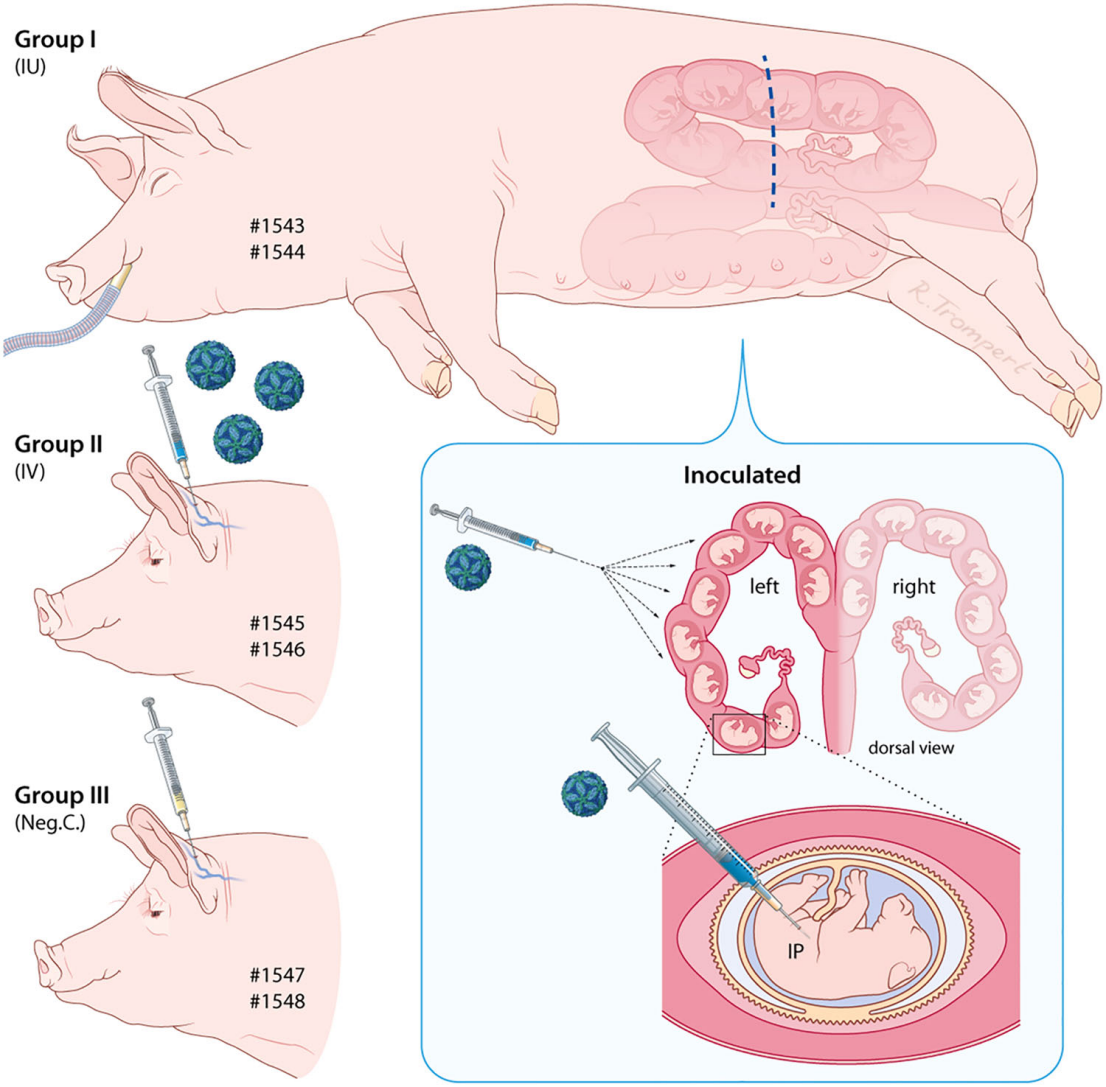
Illustration of the experimental setup. Sows 1543 and 1544 were subjected to laparotomy, after which all fetuses in the left uterine horn were inoculated, via IP route, with 10^5.6^ TCID_50_ of ZIKV strain PRVABC59. Sows 1545 and 1546 were inoculated via IV route with 10^6.4^ TCID_50_ of the same ZIKV strain and sows 1547 and 1548 were injected with culture medium, also via IV route

Sows of group II (numbers 1545 and 1546) were inoculated IV, through a catheter in the ear vein, with 10^6.4^ TCID_50_ of virus (in 1 ml) and sows of group III (1547 and 1548) were administrated (IV) with culture medium (1 ml) and served as negative controls. The experimental setup is illustrated in Fig. 1 and the operating procedure is depicted in Supplementary Figure S1. Rectal temperatures of all six sows were recorded daily. To minimize stressful conditions, which may result in abortions, blood sampling was limited to the beginning and end of the experiment. At the end of the experiment, which was 4 weeks post inoculation, all sows were humanely euthanized by IV injection of pentobarbital, followed by exsanguination. At necropsy, all fetuses and fetal organs were examined macroscopically. In addition, body and brain weights, crown-rump lengths, and head girths were measured. During necropsy, samples were collected for histology and PCR.

### ZIKV virus neutralization test (VNT)

Twofold serial dilutions of sow sera were made in microtiter plates and mixed with a fixed amount of ZIKV strain PRVABC59 (∼200 TCID_50_). After a 1.5-h incubation period, 20,000 Vero-E6 cells were added to each well containing the serum–virus mixtures. After an incubation period of 3 days at 37 °C and 5% CO_2_, plates were stained using immunoperoxidase monolayer assay (IPMA). Briefly, monolayers were fixed with 4 % (w/v) paraformaldehyde for 15 min, washed with phosphate-buffered saline (PBS), and permeabilized with ice cold methanol (100%). After washing with washing buffer (PBS, 0.05 % [v/v] Tween 20), cells were incubated for 1 h with blocking buffer (PBS, 5 % horse serum) followed by incubation for 2 h with a rabbit anti-ZIKV envelope protein primary antibody (Genetex, GTX133314) in blocking buffer. Subsequently, cells were incubated with a horseradish peroxidase-conjugated anti-rabbit IgG antibody (Dako) in blocking buffer for 1 h. Between incubations, cells were washed three times with washing buffer and antibody incubations were performed at 37 °C. Antibody binding was visualized using 3-amino-9-ethylcarbazole substrate. VNT titers were calculated using the Spearman–Kärber algorithm.

### ZIKV PCR

Organ samples were homogenized using Ultra Turrax DT-20 tubes (IKA) in cell culture medium resulting in a 10% (w/v) organ suspension. After spinning down larger cell fragments (3000 g, 10 min) RNA was isolated from the supernatant using TRIzol-LS^R^ (Invitrogen) in combination with the Direct-Zol RNA isolation kit according the manufacturer’s instructions (Zymo Research). Subsequently, after checking RNA quantity and quality on the Nanodrop, RNA was reverse-transcribed using random hexamers and Superscript III according the manufacturer’s instructions (Invitrogen). Two µl complementary DNA was subsequently used in a ZIKV-specific real-time PCR using primers ZIKV_1086_fwd and ZIKV_1162c_rev in combination with a FAM labeled probe; ZIKV_1107^2^. The PCR was performed using the LightCycler^R^ 480 Probes Master mix in a LightCycler^R^ 480 instrument as described^2^ (Roche). Data were expressed as ZIKV TCID_50_ equivalents.

### Histology and immunohistochemistry

Tissue samples were fixed in 10% phosphate-buffered formalin for 7 days, followed by routine processing into paraffin blocks. Four μm thick sections were cut and collected on silane-coated glass slides. After drying for 24 h, sections were stained with cresyl violet (Sigma) to detect Nissl substance, hematoxylin–eosin (HE) (Klinipath) or immunostained for the detection of neurofilaments. For the immunostainings, sections were pretreated for 30 min in methanol/H_2_O_2_ to block endogenous peroxidase and heated to 100 °C for 5 min in citrate buffer, pH 6 (Vector Laboratories). Sections were then incubated with a monoclonal antibody directed against swine neurofilaments (clone N52, Sigma). Mouse envision peroxidase and DAB + chromogen (Dako) was used as a secondary antibody and substrate, respectively, according the manufacturer’s instructions.

### Statistical analysis

When required data were statistically analyzed using the Mann–Whitney test in GraphPad Prism 7.0. A *P*-value of <0.05 was considered statistically significant.

## Results

### No evidence of vertical transmission of ZIKV in pregnant sows

To investigate if ZIKV is capable of crossing the porcine placenta and induces neuropathology in fetal piglets, an experiment with pregnant sows was performed. Six Landrace sows at 50 days of gestation, corresponding to almost halfway the gestation time of pigs (115 days), were randomly assigned to three groups of two animals. Sows of group I (numbers 1543 and 1544) were subjected to laparotomy, after which all fetuses in the left uterine horn were inoculated via IP route with ZIKV strain PRVABC59. In total, 20 fetuses were inoculated: 9 in the left uterine horn of sow 1543 and 11 in the left uterine horn of sow 1544. The fetuses in the right uterine horns remained untreated. Sows of group II (numbers 1545 and 1546) were inoculated via IV route with ZIKV and sows of group III (numbers 1547 and 1548) were injected via the IV route with culture medium and served as negative controls. The experimental setup is illustrated in Fig. 1 and the operating procedure is depicted in Supplementary Figure S1 and described in detail in the Materials and methods section. At 4 weeks post inoculation, which is comparable to the end of the second trimester of human pregnancy, all sows were humanely euthanized and all fetuses were examined macroscopically. In addition, body weights, crown-rump lengths, and head girths were measured and organ samples were collected (Supplementary Figure S2).

**Fig. 2:**
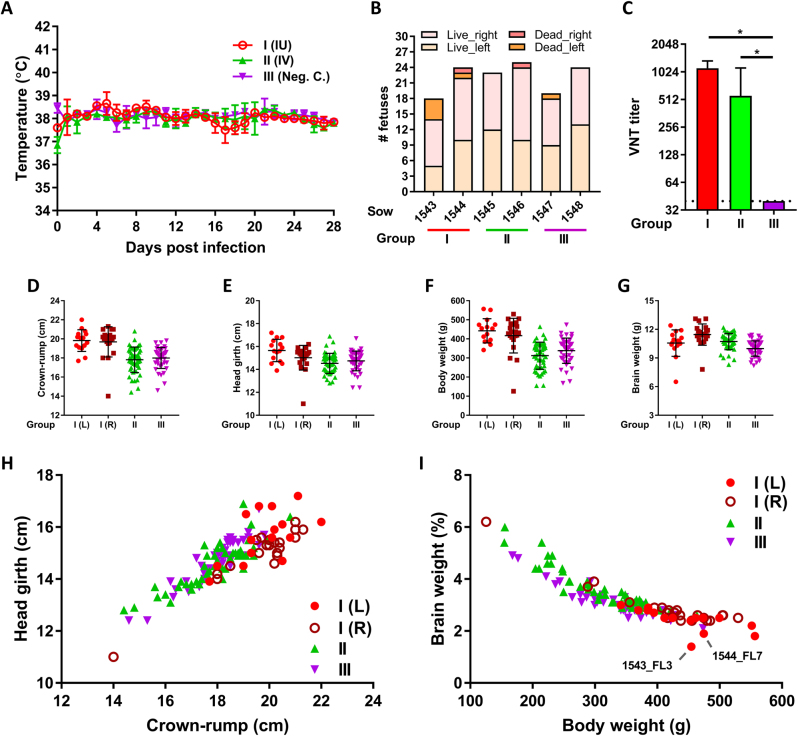
Measurements of sows and fetuses subjected to ZIKV infection. **a** Averages and standard deviations (SDs) of daily recorded sow body temperatures. **b** Numbers of live and dead (mummified) fetuses per sow and per uterine horn (left or right) at the time of necropsy. **c** ZIKV-specific VNT titers in sow serum at four weeks post challenge. Averages and SDs are presented. The limit of detection was set to 40. **d**–**g** Averages and SDs of crown-rump lengths **d**, head girths **e**, total weights **f**, and brain weights **g** of the live fetuses per experimental (sub)group. **h**, **i** Relative head size **h** and relative brain weight **i** of all individual fetuses. L left uterine horn,R right uterine horn. An asterisk indicates a statistical difference

The negative control sows (group III) did not present temperature elevation during the course of the experiment (Fig. 2a). At necropsy, sow 1547 was found to carry 18 fetuses and sow 1548 was found to carry 24 fetuses, which all appeared normal (Fig. 2b). Of note, a single mummified fetus was present in the left uterine horn of sow 1547. As expected, no macroscopic abnormalities were detected in the fetuses and all placenta and fetal brain samples were tested negative by ZIKV-specific reverse transcriptase (RT)-quantitative PCR (qPCR). Moreover, no ZIKV-specific antibodies were detected in the sera of the sows (Fig. 2c).

Like the negative control sows, the sows inoculated with ZIKV via IV route (group II) remained healthy during the course of the experiment and did not demonstrate temperature elevations (Fig. 2a). At necropsy, sows 1545 and 1546 were found to carry 23 and 24 normally developed fetuses, respectively (Fig. 2b). Sow 1546 also carried a mummified fetus. None of the normally developed fetuses demonstrated macroscopic organ abnormalities such as reduced brain growth (Figs. 2d–i). Furthermore, no viral RNA was detected in any of the sampled organs, except for a placenta sample of fetus 1546_PR8, with a Ct of 39 (1.4 TCID_50_ equivalent/g tissue). These results suggest that ZIKV strain PRVABC59 does not cross the porcine placenta, or at least not efficiently. The sows did, however, test positive for ZIKV neutralizing antibodies indicating that the infection was productive (Fig. 2c).

### Microencephaly in fetal piglets after IU inoculation of ZIKV

The sows in which the fetuses were inoculated via IP route (group I) also did not display a rise in temperature during the course of the experiment. Remarkably, neutralizing antibodies in the sera of these sows were as high as those of the IV-inoculated sows (Fig. 2c).

**Fig. 3:**
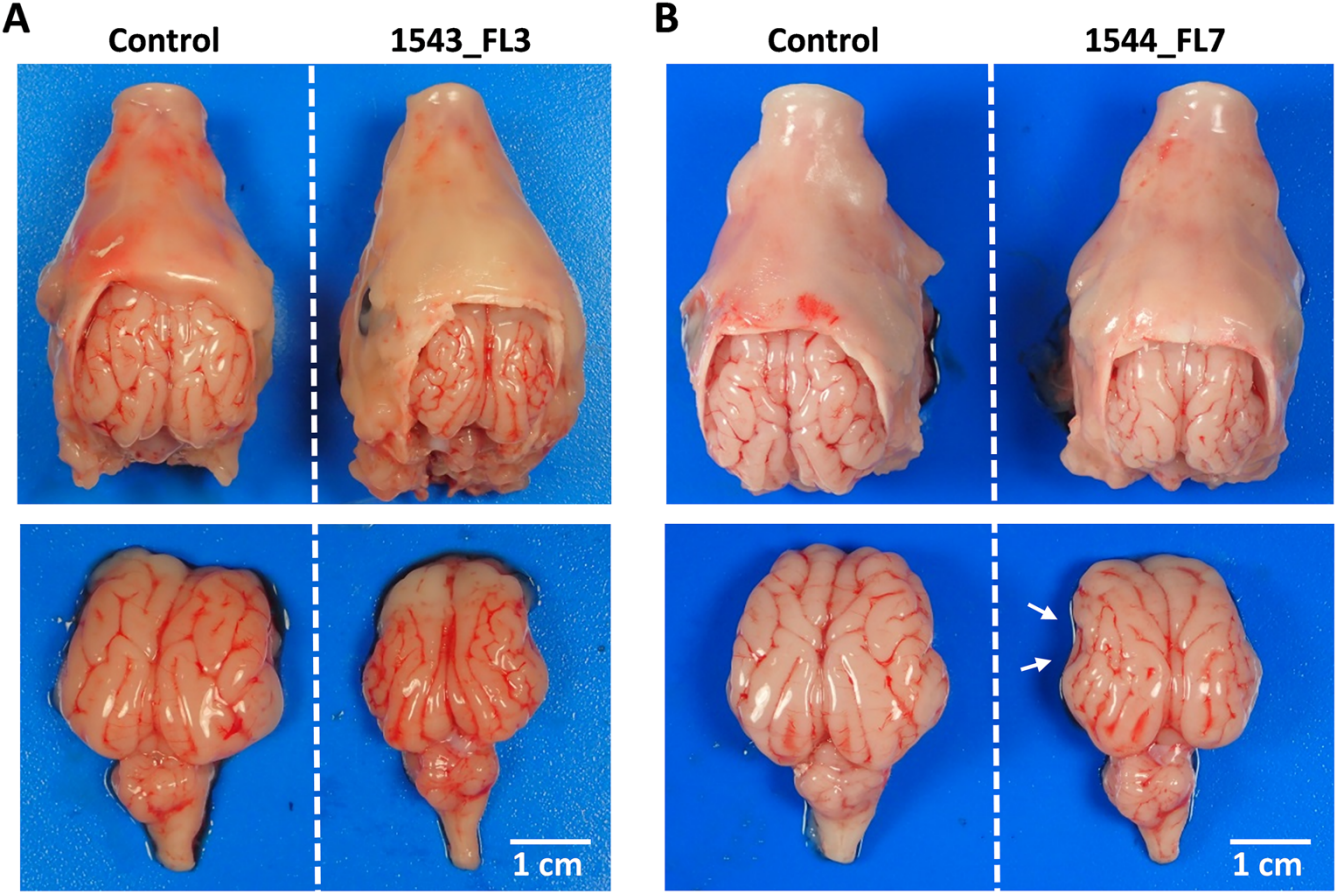
Macroscopic evidence of microencephaly in two fetal piglets following IP inoculation of ZIKV. Top view of heads and brains of fetuses presenting stunted brain growth; 1543_FL3 **a** and 1544_FL7 **b**. Note the smaller gyri of the cerebral cortex of all lobes in fetus 1543_FL3 and of the parietal, temporal, and occipital lobes in fetus 1544_FL7 (white arrows)

At necropsy, sow 1543 was found to carry 9 normally developed fetuses in the right uterine horn. In the left uterine horn, in which another 9 fetuses were inoculated with ZIKV 4 weeks earlier, 5 live fetuses and 4 mummified fetuses were found. This high number of mummified fetuses may be due to the ZIKV infection, but we cannot exclude that premature death resulted from the inoculation procedure. Interestingly, one inoculated fetus (1543_FL3) presented with microencephaly (Fig. 3a), as both cerebral hemispheres were underdeveloped with all telencephalic lobes undersized. In the other inoculated fetuses of this sow, no macroscopic abnormalities were observed. The second sow (1544) was found to carry 12 normally developed fetuses and one mummified fetus in the right uterine horn. In the left uterine horn, in which 11 fetuses were inoculated with ZIKV, 10 live fetuses and one mummified fetus were found. Importantly, also in this sow an inoculated fetus (1544_FL7) presented with microencephaly, although the brain underdevelopment was less generalized compared with fetus 1543_FL3. The parietal, temporal, and parts of the occipital lobes of 1544_FL7 were hypoplastic, whereas other lobes were of normal size (Fig. 3b). No macroscopic abnormalities were observed in the other inoculated fetuses. Although head girths of 1543_FL3 and 1544_FL7 did not differ from the other fetuses, their brain sizes were reduced and their relative brain weights were lower (Figs. 2d–i).

**Fig. 4:**
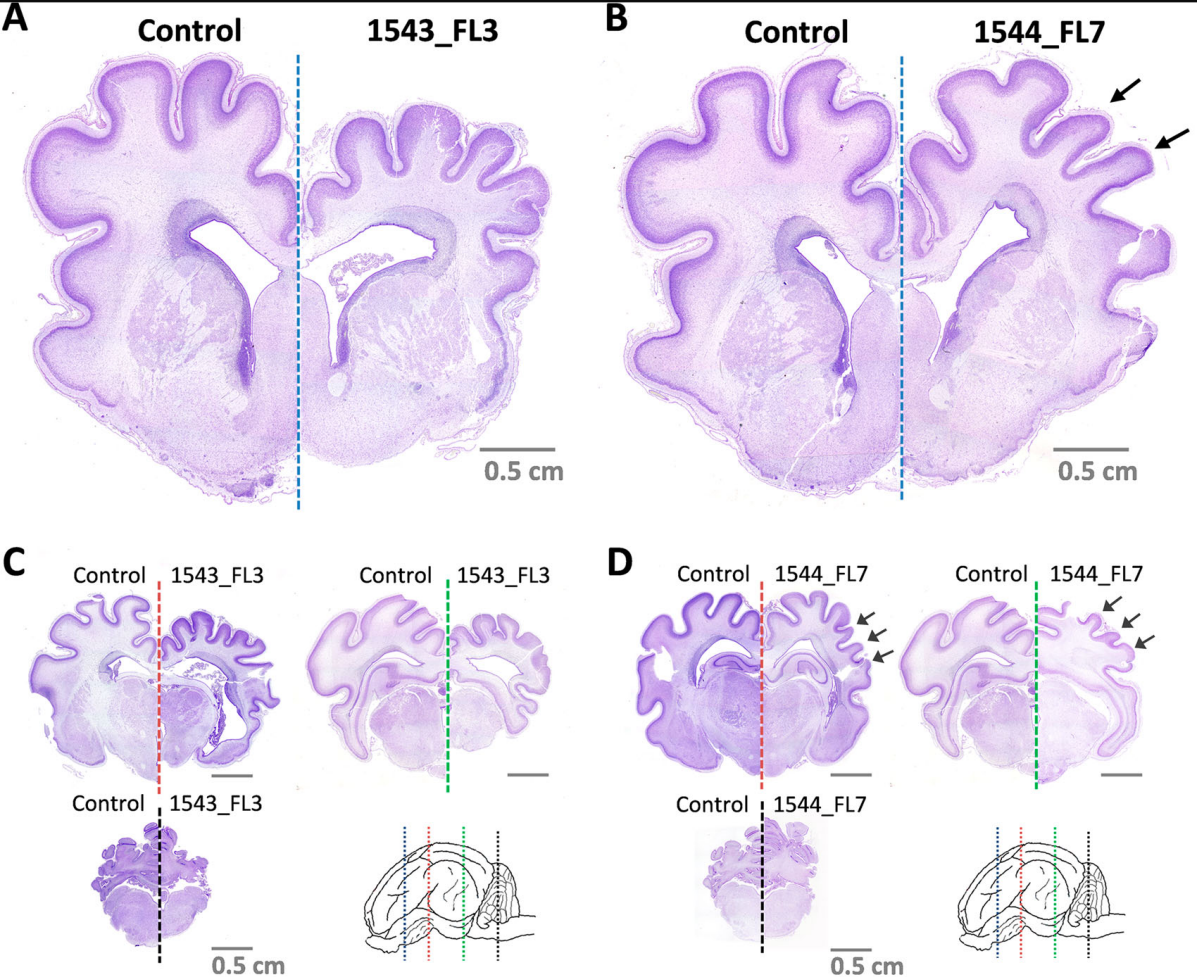
Microscopic evidence of microencephaly in 1543_FL3 and 1544_FL7. Nissl-stained brain cross-sections of 1543_FL3 **a**, **c** and 1544_FL7 **b**, **d**. Cross-sections were made of **a**,** b** the telencephalon at the striatum (blue line), **c**, **d** diencephalon at the thalamus (red line), at the transition between diencephalon and mesencephalon (green line), and at the cerebellum and medulla oblongata (black line). Arrows in** b** and **d** indicate specific underdeveloped gyri in 1544_FL7. Fetuses and brain cross-sections from the negative control group are displayed for reference

Gross histological analysis of the brains of fetus 1543_FL3 and 1544_FL7, using Nissl staining, subsequently confirmed that fetus 1543_FL3 showed a generalized microencephaly with underdevelopment of the telencephalon with all cerebral lobes affected, the diencephalon, the mesencephalon, and the metencephalon (Fig. 4), whereas in fetus 1544_FL7 only the parietal, temporal, and parts of the occipital lobes of the telencephalon were underdeveloped (Fig. 4). No histological evidence of stunted brain growth was observed in any of the other fetuses.

### ZIKV is cleared from the brain but persists in the porcine placenta

**Fig. 5:**
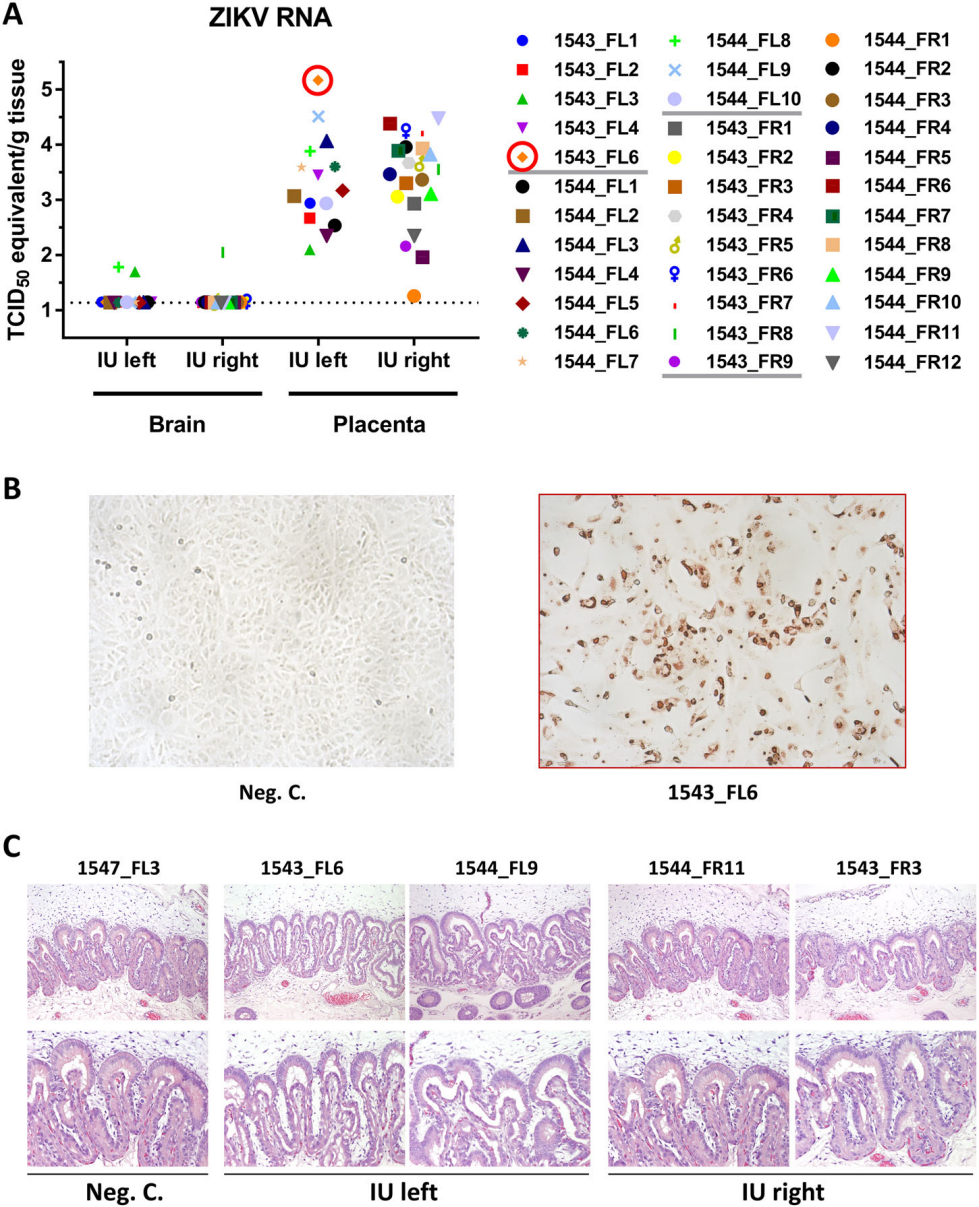
ZIKV is able to persist in pig placenta following *in utero* inoculation. **a** ZIKV-specific RT-qPCR analysis of fetal brain and placenta samples. The cut-off for the PCR was 1.14 TCID_50_ equivalent/g tissue. Infectious virus was recovered from the placenta sample from fetus 1543_FL6 (red circle). **b** IPMA of Vero cells inoculated with placenta sample 1543_FL6. **c** Representative panel of HE-stained placenta samples derived from the IU-inoculated sows

ZIKV-specific RT-qPCR analysis of fetuses collected from IU-inoculated sows subsequently detected only very low levels of ZIKV RNA in the brains of three fetuses; 1543_FL3 included (Fig. 5a). The absence of ZIKV in most brain samples 4 weeks post inoculation may be explained by apoptosis or necrosis of virus-infected cells or by the induction of an effective immune response.

In contrast to the brain samples, PCR analysis of samples obtained from the placentas demonstrated that ZIKV persisted in the placentas of the IU-inoculated sows. Strikingly, in placenta samples collected from the right uterine horns, in which no fetuses were inoculated, similar levels of ZIKV RNA were detected. This may be the result of direct spreading of the virus from the left to the right uterine horn or, alternatively, from dispersion via maternal blood. Despite the relatively high levels of ZIKV RNA in the placentas, HE staining did not reveal any histological abnormalities (Fig. 5c). Notably, infectious virus was recovered from the placenta sample of fetus 1543_FL6, which also contained the highest level of viral RNA (Fig. 5b).

### Mild to severe neuronal depletion in fetal brains following ZIKV inoculation

**Fig. 6:**
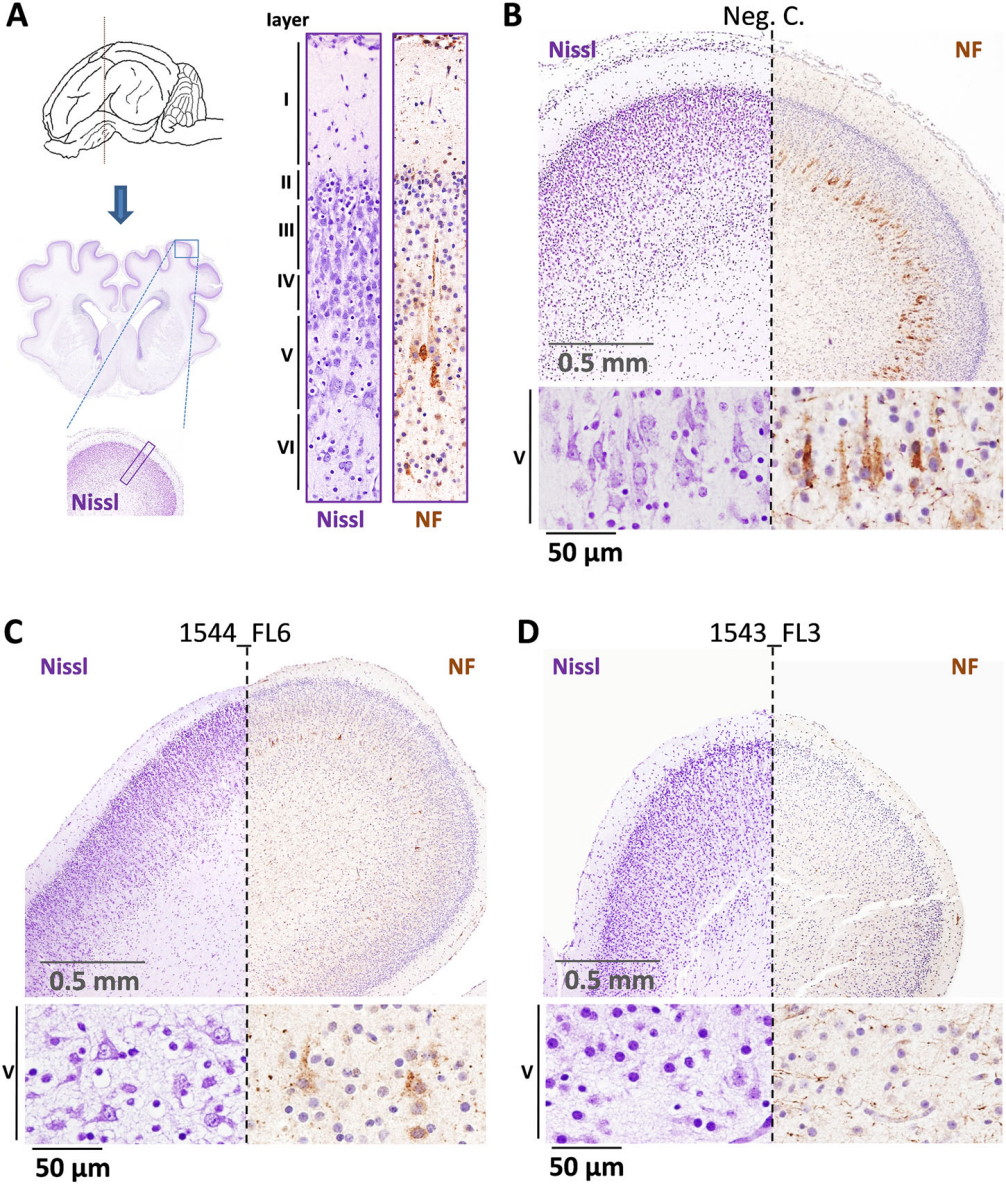
Neurodepletion in the parietal lobe following IU inoculation of ZIKV. **a** Overview of the localization of the parietal lobe and its cytoarchitecture in the normal fetal pig brain. The different cortical layers (I–VI) are indicated. **b** Images of Nissl and neurofilament (NF)-stained parietal lobe cross-sections of a negative control fetus and a higher magnification of the pyramidal neurons of cortical layer V. **c** A fetus presenting moderate neurodepletion in cortical layer V (1544_FL6) and **d** a fetus presenting severe neurodepletion in cortical layer V with prominent lobe hypoplasia (1543_FL3)

More detailed analysis of the underdeveloped brains of fetuses 1543_FL3 and 1544_FL7 revealed a severe reduction to almost complete absence of cortical neurons in layers III and V, corresponding to the external and internal pyramidal layer, respectively, and layer VI, corresponding to the multiform or fusiform layer. Immunohistochemistry using antibodies directed against neurofilaments confirmed the depletion of pyramidal neurons in layer V (Fig. 6) with concurrent hypoplasia of descending fibers in the cortical white matter tracts.

**Fig. 7:**
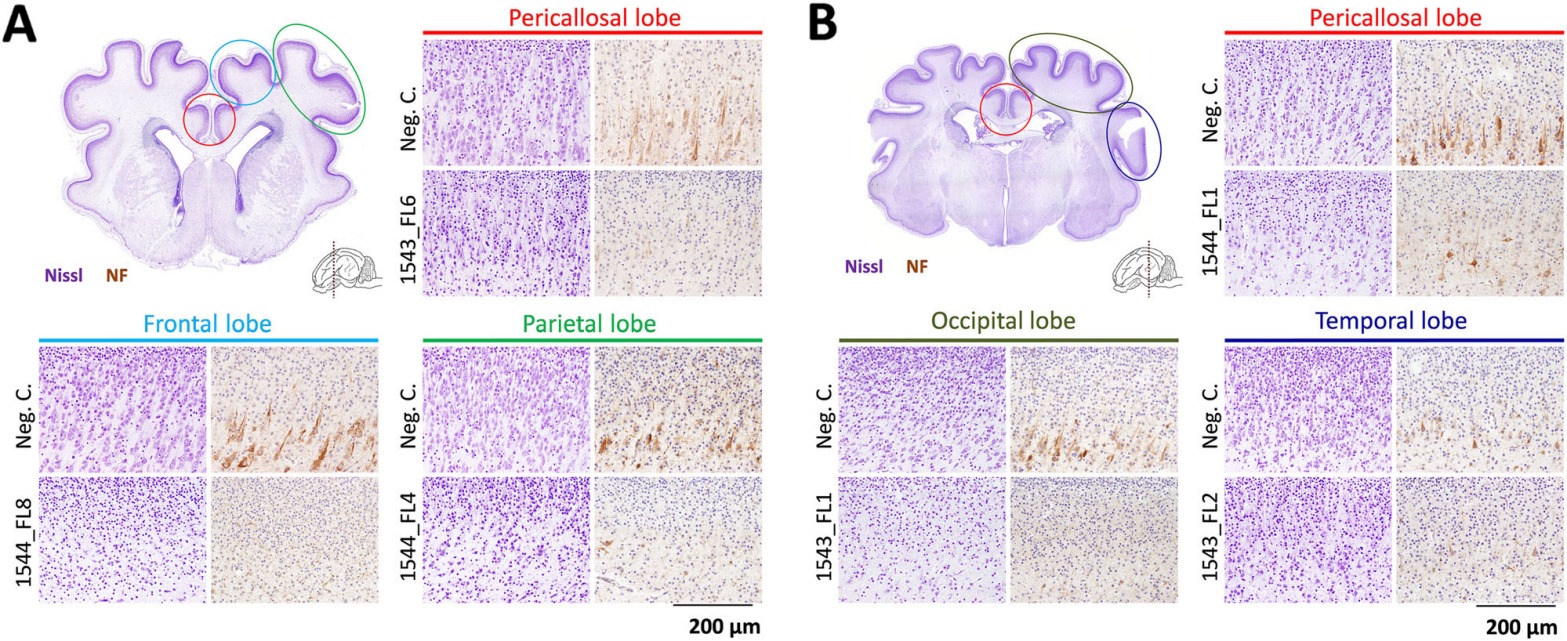
Neurodepletion in all other lobes following IU inoculation of ZIKV. Nissl and neurofilament (NF)-stained cross-sections of the cerebral cortical lobes at the striatum **a** and thalamus **b** of other inoculated fetuses presenting neurodepletion. The various cerebral lobes are indicated. For reference, images are included which are derived from similar cross-sections and from the same cerebral lobes of negative control fetuses. NF neurofilament

Importantly, localized neuronal depletion was also demonstrated in the cerebral cortices of various lobes of all other inoculated fetuses, despite the fact that none of these fetuses presented any macroscopic evidence of cerebral lobe hypoplasia or microencephaly (Fig. 7). A similar neurodepletion was visible in the cerebral lobes of fetus 1544_FL7 that did not show macroscopic signs of hypoplasia. No histological abnormalities were observed in the brains of any of the fetuses from the right uterine horn and in brains of fetuses obtained from the IV-inoculated sows.

## Discussion

The recent ZIKV epidemic has urged the development of mouse and nonhuman primate models. Both types of models have contributed greatly to our understanding of ZIKV-induced neuropathology. Although nonhuman primates most closely resemble humans in development and physiology, their use in large numbers raises ethical, practical, and financial issues. The availability of an additional model, preferably based on another large mammal, could further benefit and speed up ZIKV research. We here evaluated whether ZIKV-mediated neuropathology as observed in human neonates can be studied in pigs.

A previous study with neonatal piglets showed that ZIKV is able to induce neurological disease after inoculation of the virus directly into the brain^26^. Interestingly, no evidence of disease or pathology was reported after peripheral administration of the virus, suggesting that ZIKV does not cross the blood–brain barrier (BBB) of newborn piglets. In the present work, the observed mild to severe neuropathology in 100% of IP-inoculated fetuses demonstrates that ZIKV is capable of crossing the BBB very efficiently in the developing fetus. To what extent the amount of ZIKV susceptible cells and the integrity and permeability of the BBB, which may differ between pig fetuses and neonates, influences ZIKV infection awaits further study.

Despite the clear lesions in the brain, no infectious virus, nor high levels of viral RNA, were detected in corresponding brain samples collected 4 weeks post inoculation, thereby preventing the identification of primary target cells. Nevertheless, numerous studies already showed that neural progenitors are the primary ZIKV target cells in the fetal brain^27, 28^. Thus, the observed depletion of mature neurons in the inoculated pig fetuses, as evidence by a strong reduction in neurofilament positive cells, is expected to be the direct result of ZIKV-induced depletion of neural progenitors. We hypothesize that by focusing on earlier time points in infection, the presented model offers an ideal tool to characterize the course of ZIKV dissemination to and in the fetal brain, and thereby offers the possibility to identify important host and virus factors involved. Moreover, such studies could reveal whether ZIKV-infected cells were removed from the brain by apoptosis or necrosis, or alternatively, were cleared by the fetal immune system.

Two out of 15 inoculated fetal piglets were found to have a smaller than average brain, which is known as microencephaly. In human fetuses, a smaller brain is generally accompanied by a reduced head size, referred to as microcephaly, which is considered the most profound clinical sign of congenital ZIKV syndrome. Of note, in literature the term microcephaly is not exclusively used for a smaller then average skull but also for a smaller than average brain in the absence of skull abnormalities. In our study, the reduced brain size was not accompanied by a reduced head size, which may be explained by differences in physiology and development of the human and porcine skull. In accordance with this proposition, microcephaly in pigs has not been reported as far as the authors are aware.

Infectious ZIKV or viral RNA was detected in all placenta samples of the inoculated fetuses 4 weeks post inoculation. Apparently, and similar as observed in humans, ZIKV can persist in the porcine placenta. Samples obtained from the right uterine horn, in which no fetuses were inoculated, were found to contain comparable levels of viral RNA as well. This finding may be explained by direct transmission of ZIKV between uterine horns or, alternatively, by systemic spreading of the virus. The relatively high levels of neutralizing antibodies detected in corresponding blood samples may be indicative of the latter. Similar as in humans, no necrosis and leukocyte infiltrations were detected by histopathology in the placenta samples. However, it must be noted that in humans, enlarged hydropic chorionic villi with hyperplasia and focal proliferation of macrophages were observed, which we did not observe in the present study^29–31^.

Although ZIKV-mediated neuropathology was observed in all inoculated fetuses, no signs of disease were detected in any of the fetuses from the IV-inoculated sows or in the fetuses from the right uterine horn of the IU-inoculated animals. This suggests that ZIKV is unable to cross the porcine placenta or that the systemic ZIKV load is too low to support vertical transmission. To improve both the IV and the IU model, it would be valuable to use pig passaged ZIKV as an inoculum. Furthermore, it would be interesting to test additional ZIKV strains besides PRVABC59^32^. Differences in transmission efficiency and pathology between different ZIKV strains is well known^10, 33–35^. Finally, it would be interesting to inoculate the sows and fetuses, although practically more challenging, earlier in gestation as neuropathology in human neonates is mainly associated with a ZIKV infection during the first trimester of pregnancy^36, 37^.

In the course of finalizing this article, a related study was published in which pig fetuses were inoculated with ZIKV^38^. This study confirmed several of our findings, such as the susceptibility of porcine fetuses to ZIKV infection and the ability of ZIKV to persist in the porcine placenta. Interestingly, the authors provided preliminary evidence that IU-inoculated fetuses with normal brain sizes present neurological manifestations after birth. Importantly, the authors did not study the integrity of the cerebral cortex as we describe in the present work. The combined results strongly suggest that ZIKV infection of porcine fetuses induces health impairments resulting from depletion of cortical neurons. The latter also supports the notion that congenital ZIKV infection induces more than just microcephaly^28, 39–41^.

Altogether, the fetal pig model can be used to study fundamental aspects of congenital ZIKV syndrome and may facilitate the evaluation of ZIKV therapeutics.

## Electronic supplementary material

Supplementary Figure 1

Supplementary Figure 2
